# Deep Learning in the Detection and Diagnosis of COVID-19 Using Radiology Modalities: A Systematic Review

**DOI:** 10.1155/2021/6677314

**Published:** 2021-03-15

**Authors:** Mustafa Ghaderzadeh, Farkhondeh Asadi

**Affiliations:** ^1^Student Research Committee, Department and Faculty of Health Information Technology and Management, School of Allied Medical Sciences, Shahid Beheshti University of Medical Sciences, Tehran, Iran; ^2^Department of Health Information Technology and Management, School of Allied Medical Sciences, Shahid Beheshti University of Medical Sciences, Tehran, Iran

## Abstract

**Introduction:**

The early detection and diagnosis of COVID-19 and the accurate separation of non-COVID-19 cases at the lowest cost and in the early stages of the disease are among the main challenges in the current COVID-19 pandemic. Concerning the novelty of the disease, diagnostic methods based on radiological images suffer from shortcomings despite their many applications in diagnostic centers. Accordingly, medical and computer researchers tend to use machine-learning models to analyze radiology images. *Material and Methods*. The present systematic review was conducted by searching the three databases of PubMed, Scopus, and Web of Science from November 1, 2019, to July 20, 2020, based on a search strategy. A total of 168 articles were extracted and, by applying the inclusion and exclusion criteria, 37 articles were selected as the research population.

**Result:**

This review study provides an overview of the current state of all models for the detection and diagnosis of COVID-19 through radiology modalities and their processing based on deep learning. According to the findings, deep learning-based models have an extraordinary capacity to offer an accurate and efficient system for the detection and diagnosis of COVID-19, the use of which in the processing of modalities would lead to a significant increase in sensitivity and specificity values.

**Conclusion:**

The application of deep learning in the field of COVID-19 radiologic image processing reduces false-positive and negative errors in the detection and diagnosis of this disease and offers a unique opportunity to provide fast, cheap, and safe diagnostic services to patients.

## 1. Introduction

With the outbreak of an unknown disease in late 2019 in China, some people became infected with the disease in a local market. The disease was completely unknown at first, but specialists diagnosed its symptoms as similar to those of coronavirus infection and flu [1–4]. The specific cause of this widespread disease was initially unknown, but after the laboratory examination and analysis of positive sputum by real-time polymerase chain reaction (PCR) test, the viral infection was confirmed and eventually named “COVID-19” upon the recommendation of the World Health Organization (WHO). Over a short period, the COVID-19 epidemic crossed geographical boundaries with a devastating effect on the health, economy, and welfare of the global population [1, 5]. Based on the Worldometers (worldometers.info) statistics, until January 5, 2021, more than 86 million people worldwide contracted COVID-19, of whom more than 1,870,000 people died officially due to the disease. The early detection of COVID-19 is essential not only for patient care but also for public health by ensuring the patients' isolation and controlling the pandemic [6–8]. Due to the novelty of the disease, ways to fight it were not known in the early days, but researchers considered screening and rapid diagnosis of infected patients and their separation from the community of healthy people as an important measure. The clinical features of COVID-19 include respiratory symptoms, fever, cough, dyspnea, and pneumonia. However, these symptoms do not always indicate COVID-19 and are observed in many cases of pneumonia, leading to diagnostic problems for physicians [2, 6, 9].

While the RT-PCR test is the gold standard for diagnosing COVID-19, it has limiting aspects with certain features that make it difficult to diagnose the disease. RT-PCR is a very time-consuming, complex, costly, and manual process. One of the drawbacks of this method is the need for a laboratory kit, the provision of which is difficult or even impossible for many countries during crises and epidemics. Like all diagnostic and laboratory methods in healthcare systems, this method is not error-free and is biased. It requires an expert laboratory technician to sample the nasal and throat mucosa which is a painful method, and this is why many people refuse to undergo nasal swap sampling [10–13]. More importantly, many studies indicated the low sensitivity of the RT-PCR test; several studies have reported the sensitivity of this diagnostic method to be 30% to 60%, indicating a decrease in the accuracy of the diagnosis of COVID-19 in many cases. Some studies also pointed to its false-negative rate and contradictory results [14, 15].

One of the most important ways to diagnose COVID-19 is to use radiological images, including X-ray and computed tomography (CT) scan. Chest imaging is a quick and easy procedure recommended by medical and health protocols and has been mentioned in several texts as the first tool in screening during epidemics [16, 17].

Compared to RT-PCR, CT scan images have a high sensitivity in diagnosing and detecting cases with COVID-19; however, their specificity is low. This means that CT scan is more accurate in cases of COVID-19, but less accurate in cases of nonviral pneumonia. A study conducted on the diagnosis of patients in Wuhan, China, showed that consolidation and ground-glass opacities (GGO) were not observed in CT scan imaging in 14% of the images, meaning that 14% of the definitive cases of COVID-19 were misdiagnosed as completely healthy based on their CT scan tests. Out of 18 patients with COVID-19 who had GGO with consolidation, only 12 had GGO and, as a result, no consolidation or disease was observed. Despite the presence of consolidation without the advent of GGO in many cases, it was difficult and almost impossible to detect COVID-19. All these cases demonstrated a defect in the diagnosis of COVID-19 using CT scans [18–21].

Despite the success of chest CT scan in detecting COVID-19-related lung damage, certain problems are associated with the use of this diagnostic test. Despite the WHO's recommendation, chest CT findings are normal in some patients at the outset of the disease, and this makes the use of CT alone to have a negative predictive value. The low specificity of CT scan can cause problems in the detection of non-COVID-19 cases. In addition, the CT scanner rays can cause problems for patients who require multiple CT scans during the course of the disease. The American College of Radiology recommends that CT scans should not be used as the first line of diagnosis. Problems such as the risk of transmission of the disease while using a CT scan device and its high cost can cause serious complications for the patient and healthcare systems, so it is recommended that if medical imaging is needed, the CT scan be replaced with CXR radiography [22]. X-ray imaging is much more extensive and cost-effective than conventional diagnostic tests. Transmission of an X-ray digital image does not require transferring from the access point to the analysis point, so the diagnostic process is performed very quickly. Chest radiography is convenient and fast for medical triaging of patients. Unlike CT scans, X-ray imaging requires less scarce and expensive equipment, so significant savings can be made in the running costs. Furthermore, portable CXR devices can be used in isolated rooms to reduce the risk of infection resulting from the use of these devices in hospitals [23, 24]. Various studies have indicated the failure of CXR imaging in diagnosing COVID-19 and differentiating it from other types of pneumonia. The radiologist cannot use X-rays to detect pleural effusion and determine the volume involved. However, regardless of the low accuracy of X-ray diagnosis of COVID-19, it has some strong points [25, 26]. To overcome the limitations of COVID-19 diagnostic tests using radiological images, various studies have been conducted on the use of deep learning (DL) in the analysis of radiological images.

## 2. Materials and Methods

### 2.1. Deep Learning

In 2006, Hinton and Salakhutdinov published an article in the Science journal that was a gateway to the age of DL. They showed that a neural network with hidden layers played a key role in increasing the learning power of features. These algorithms can enhance the accuracy of classifying different types of data [27]. One of the major applications of DL in radiology practices was the detection of tissue-skeletal abnormalities and the classification of diseases. The convolutional neural network has proven to be one of the most important DL algorithms and the most effective technique in detecting abnormalities and pathologies in chest radiographs [28]. Since the outbreak of COVID-19, much research has been conducted on processing the data related to DL algorithms, especially CNN. Using different algorithms and DL architectures, these studies have embarked on the identification and differential diagnosis of COVID-19. Herein, these studies have been systematically analyzed. This study was accomplished by a structured review method to identify studies related to the identification and diagnosis of COVID-19. A systematic search strategy was developed by using previous studies and the authors' opinions.

### 2.2. Search Criteria

1. To what extent has the use of DL been able to improve the routine methods of diagnosing COVID-19?
2. What modalities can be used to help identify and diagnose COVID-19 by using DL?
3. Has DL been able to cover the shortcomings of diagnostic modalities?
4. How is the efficacy of different types of DL and its architectures in promoting the diagnosis of COVID-19 compared to one another?

The researchers reviewed electronic databases to identify studies on medicine and computer sciences and concluded that PubMed, Web of Science, and Scopus contain the highest number of publications related to the present study. The following key terms were used as the search strategy: “COVID-19,” “diagnosis,” “detection,” and “deep learning” from November 1, 2019, to July 20, 2020, and related published studies were extracted from the three databases. The EMBASE and IEEE databases were removed from the search domain due to the similarity of their publications.

### 2.3. Data Extraction

**Figure 1:**
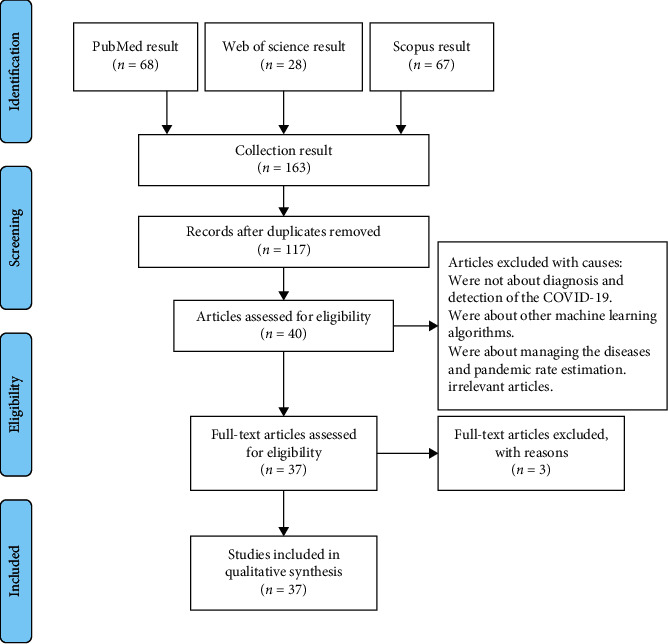
PRISMA flow diagram of the review process and exclusion of papers.

Relevant studies, details of their methodologies, and their results were recorded in data extraction forms. Data selection and extraction were performed based on Figure 1. To identify algorithms and DL methods, the main details of the methods and their results were recorded in data extraction sheets. Two researchers (F.A. and M.Q.) extracted the data, and differences between the studies were resolved through discussions. The extracted data elements included the name of the study, country, year of publication, research population, modality, data used, DL techniques, evaluation methods, and results.

## 3. Results

Initially, 160 abstracts and full-text articles were assessed, and ultimately, 37 studies meeting the inclusion criteria were selected. The PRISMA method was adopted in the process of selecting the articles. Due to the novelty of the disease, all the selected articles were published in 2020. Out of 37 extracted articles, eight articles were published in India, five in China, five in the United States, and three in Turkey. Moreover, Iran, Greece, Italy, and Egypt each presented two research papers, and Morocco, Bangladesh, Spain, Colombia, Iraq, Brazil, Canada, and South Korea each presented one study in this field.

### 3.1. Purpose of Deep Learning in the Analysis of Radiology Images about COVID-19

Image-based diagnostic methods in epidemics play a key role in the screening of affected cases. CXR and CT scan are among the main radiology modalities in the detection and diagnosis of COVID-19. In all the studies reviewed here, radiological images have been analyzed to diagnose COVID-19 with DL. This study was conducted around two common terms, “detection” and “diagnosis,” to characterize the presence of COVID-19. Collins Dictionary defines the terms “detection” and “diagnosis” differently from the medical point of view. However, in the field of medical image analysis, these two terms have been used interchangeably. By examining the existing texts and dictionaries and seeking advice from radiologists and epidemiologists, detection is defined as part of the real entity that can be seen or whose existence can be proved or disproved. In medical texts, detection is considered as a prelude to diagnosis. Similarly, in the case of COVID-19, many studies have used these two words interchangeably, but they are clinically different from each other. By distinguishing these two terms from each other, detection was considered in this study as distinguishing the cases infected with COVID-19 from non-COVID-19 ones. This means that no information is available on the type of the disease in non-COVID-19 patients, and this group can have different types of bacterial pneumonia, viral, or other groups of coronavirus diseases with the exception of COVID-19. We also considered diagnosis as a term to distinguish COVID-19 from other infectious lung diseases such as different types of pneumonia. Diagnosis is meaningful in categories where the rest of the diseases (not infected with COVID-19) are well-specified, and COVID-19 can be distinguished with certainty from types of pneumonia or other coronaviruses. In this regard, by examining the extracted articles, it was found that 15 articles had used DL to detect (identify) COVID-19 [29–40].

**Figure 2:**
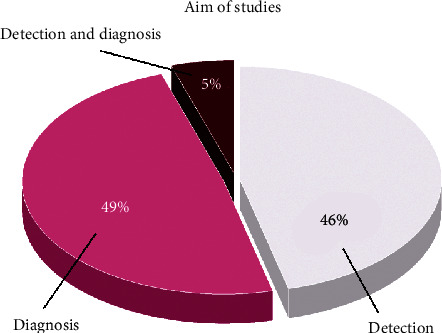
Aim of studies in processing of COVID-19 radiology modalities by means of DL.

On the other hand, many articles have diagnosed COVID-19 with DL algorithms [41–56]. In these cases, COVID-19 was accurately diagnosed among the different types of pneumonia. Some studies have analyzed radiology modalities to detect and diagnose it simultaneously [31, 57].  Figure 2 displays the studies on the detection and diagnosis of COVID-19. As noted earlier, a diagnostic disadvantage of CT scan images in identification of cases of COVID-19 is its low specificity. This investigation found that many studies have attempted to improve these methods in the analysis of CT scan images with DL techniques [58, 59, 9]. Apparently, these methods owe their success in finding pulmonary lesions caused by COVID-19 to the extraction and selection of features hidden in the images. Despite the improvement in the detection and diagnosis of COVID-19 by DL algorithms, one of the biggest drawbacks of this modality in the diagnosis of COVID-19 was the lack of this equipment in all medical and diagnostic centers. Furthermore, many patients with COVID-19 required multiple chest images using CT scans. Exposure to radiation during CT scans causes serious problems for patients. Moreover, there is a danger of transmission of the virus from a patient to others due to CT scan tunnel contamination.

**Figure 3:**
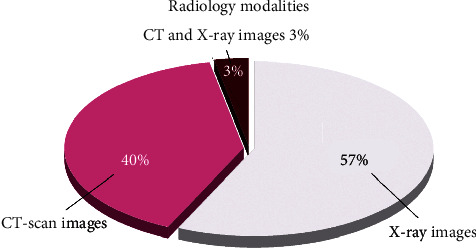
Rate of using different radiological modalities in processing of COVID-19 by means of DL.

Therefore, many researchers and physicians have resorted to plain radiographic images or X-rays to diagnose COVID-19. Nevertheless, these images do not have the necessary resolution and accuracy in diagnosing COVID-19 from the beginning and have many disadvantages in this regard. Therefore, artificial intelligence researchers rushed to the help of clinical experts and used DL as a powerful tool to improve the accuracy of the diagnosis of COVID-19 with X-ray images [60]. Due to the nature of DL in the extraction of image features, this technology is capable of detecting patients with COVID-19 and extracting infectious lung tissues, so many studies embarked on a variety of DL algorithms to analyze these images [32, 36, 38, 40, 54, 59, 61]. In the early days of the COVID-19 outbreak, CT scans were more common in its diagnosis, but over time, X-rays also became common. Thus, research also shifted from CT scan image analysis to radiographic image analysis.  Figure 3 illustrates the degree of analysis of the two modalities used to detect and diagnose COVID-19.

**Figure 4:**
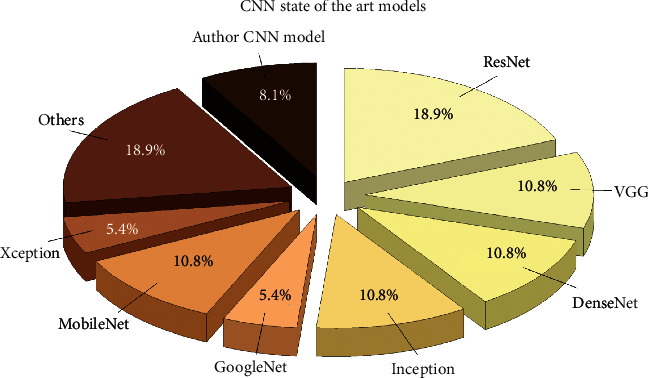
Rate of CNN architectures used in the analysis of radiology modality images of COVID-19.

One of the main features of deep neural networks, in terms of their efficacy, is their employed architecture. Deep neural network architectures demonstrate an extraordinary ability to perform a variety of functions for different data types. Various studies have been conducted on COVID-19 with different DL architectures. In a number of these studies, their diagnosis rate was compared in the detection of COVID-19 by using different types of architectures [58]. The frequency of CNN architectures used in the reviewed studies can be seen in Figure 4. The architectures presented in this figure represent a family of the same architectures or different editions of that architecture. In the texts reviewed, the ResNet architecture achieved the most efficacy. However, some of the studies with ResNet-50 architecture achieved the best efficacy in detecting and diagnosing COVID-19, and others utilized other ResNet editions to maximize efficacy in analyzing radiological images for the diagnosis of COVID-19. It was found that newer and more developed architectures were more efficient in the diagnosis of COVID-19.

### 3.2. Deep Learning Techniques in the Detection and Diagnosis of COVID-19

Studies suggest that different DL techniques have been adopted for the detection, diagnosis, classification, prediction, and prognosis of COVID-19. Domestic datasets (including CT and X-ray images) or public datasets have been employed in some studies in which training and testing datasets were used to train and validate the methods. The criteria for measuring the efficiency of methods used in community studies include sensitivity, specificity, and accuracy. Nevertheless, in many studies, AUC has also been used to determine the efficiency of the method used to diagnose COVID-19.

In a number of studies, the proposed method has been implemented based on well-known or state-of-the-art architectures. However, some studies have also presented their own customized algorithm and architecture, independent of well-known architectures (Table 1).

## 4. Discussion

This systematic review evaluated 37 studies in order to assist researchers to explore and develop knowledge-based systems based on artificial intelligence in the detection and diagnosis of COVID-19. To the best of our knowledge, the current review, which reviewed a variety of DL methods to analyze radiological images, is one of the most comprehensive studies on the diagnosis and detection of this disease. The current review provided up-to-date information on DL algorithms and their application as an expression of radiographic imaging analysis of COVID-19. Many studies have shown that the use of DL algorithms can improve the rate of metric features of CT scan images and enhance the sensitivity and specificity of radiographic images compared to the radiologists' diagnosis; therefore, the use of this inexpensive and affordable modality should be considered as a reliable method for the diagnosis of COVID-19. By reviewing 23 research papers on the application of X-ray in the diagnosis of COVID-19 by using DL methods, the current modality can be introduced to the scientific and medical community for the early and rapid diagnosis of this disease. By improving imaging methods through artificial intelligence technologies, we can find the cheapest and safest imaging methods to prevent the transmission of COVID-19. A review of published studies showed that the diagnosis of this disease by DL algorithms under the supervision of a radiologist led to improved efficacy and reduced diagnostic errors in various cases of pneumonia, especially COVID-19. The mean diagnosis of all the studies using the X-ray modality had a sensitivity average >95%, a specificity >91%, and a higher rate of diagnosis than that reported in traditional texts and methods.

It can also be concluded that the specificity in CT scan images obtained by the DL method in case of COVID-19 was on average higher than 92% which, in many cases, has higher efficiency in terms of specificity compared to previous texts. The sensitivity of DL methods in CT scan images of COVID-19 was also higher than or equal to that of the usual diagnostic methods in many cases. Due to the excessive similarity of the effects of COVID-19 on lung tissue with different types of bacterial and viral pneumonia, the diagnosis of these diseases through unsupervised methods is very difficult and complicated. The examination of the algorithms and DL architectures revealed that almost all the studies have utilized the CNN algorithm; of course, other algorithms have also been used along with the CNN algorithm in other studies. The CNN architectures employed in these studies all have special features in image analysis, and without adjusting their parameters, it is not possible to have access to the ability of these architectures to detect and diagnose COVID-19.

## 5. Conclusion

As discussed before, the early detection and diagnosis of COVID-19 by DL techniques and with the least cost and complications are the basic steps in preventing the disease and the progression of the pandemic. In the near future, with the incorporation of DL algorithms in the equipment of radiology centers, it will be possible to achieve a faster, cheaper, and safer diagnosis of this disease. The use of these techniques in rapid diagnostic decision-making of COVID-19 can be a powerful tool for radiologists to reduce human error and can assist them to make decisions in critical conditions and at the peak of the disease. This research supports the idea that DL algorithms are a promising way for optimizing healthcare and improving the results of diagnostic and therapeutic procedures. Although DL is one of the most powerful computing tools in diagnosis of pneumonia, especially COVID-19, developers should be careful to avoid overfitting and to maximize the generalizability and usefulness of COVID-19 DL diagnostic models; these models must be trained on large, heterogeneous datasets to cover all the available data space.

## Figures and Tables

**Table 1:** Studies evaluating deep learning algorithms used for COVID-19 detection and diagnosis.

Reference (country)	Aim of the study	Population	Feature engineering	ML method	Model	Type of data	Validation results
Saiz and Barandiaran, [62] (Spain)	Detection	1500	Automatic feature extraction	CNN with transfer learning	VGG-16 SDD	X-ray	Accuracy: 94.92%, sensitivity: 94.92%, specificity: 92%
Ni et al., [34] (China)	Detection	14531	Prominent features selected	Deep learning	Convolutional MVP-Net and 3D U-Net	CT images	F1 score: 97% in detecting lesions, sensitivity: 100%, for detecting patient sensitivity for per-lung Lobe lesion: 0.96%
Wang et al., [21] (China)	Diagnosis and prognosis	5372 (two datasets)	Not used for diagnosis	Deep learning	DenseNet121-FPN	CT images	AUC = 87% and 88%, sensitivity: 80.3% and 79.35%, specificity: 76.3% and 81.1%
Rahimzadeh & Attar, [50] (Iran)	Diagnosis	11302 images (open source)	Automatic feature extraction	Deep learning	Xception and ResNet50V2	X-ray	Accuracy: 95.5%, overall average accuracy: 91.4%
Panwar et al., [36] (India)	Fast detection	337 images (open source)	Not used	Deep learning (nCOVnet)	VGG-16	X-ray	Sensitivity: 97.62%, specificity: 78.57%, accuracy: 88.10%
Ardakani et al., [30] (Iran)	Detection	194	Not used	Deep learning	AlexNet, VGG-16, VGG-19, SqueezeNet, GoogLeNet, etc.	CT images	Sensitivity: 100%, specificity: 99.02%, accuracy: 99.51%
Li et al., [46] (China)	Diagnosis	4356 CT exams from 3322 patients	Automatic feature extraction	Deep learning	ResNet-50 as backbone of main model	CT images	Sensitivity: 90%, specificity: 96%
Li et al., [19] (Greece)	Automatic diagnosis	2914	Automatic feature extraction	CNN with transfer learning	MobileNetV2	X-ray	Accuracy: 96.78%, sensitivity: 98.66%, specificity: 96.46%
Sethy et al., [51] (India)	Diagnosis	381	Automatic feature extraction	CNN and SVM	ResNet-50	X-ray	Sensitivity: 95.33%
Song et al., [37] (Chain and USA)	Detection	227	Automatic feature extraction	Deep learning (CoroNet)	BigBiGAN^1^	CT images	Sensitivity: 85%, specificity: 88%
Brunese et al., [31] (Italy)	Detection	6,523	Automatic feature extraction	Deep learning (CoroNet)	VGG-16	X-ray	Accuracy: 97%
Butt et al., [42] (USA)	Classification (diagnosis)	618	Automatic feature extraction	CNN	ResNet-18	CT images	Sensitivity: 98.2%, specificity: 92.2%
Loey and et al., [63] (Egypt)	Diagnosis (classification)	306	Automatic feature extraction	Deep learning	GoogLeNet	X-ray	Accuracy: 100%
Ozturk et al., [35] (Turkey)	Automated detection	2 databases	Automatic feature extraction	Deep learning	DarkNet	X-ray	Binary case accuracy: 98.08%, multiclass cases accuracy: 87.02%
El Asnaoui and Chawki, [58] (Morocco)	Diagnosis	6087	Automatic	Deep learning	Inception_ResNet_V2	X-ray and CT	Inception_ResNet_V2 accuracy: 92.18%, DenseNet201 accuracy: 88.09%
Yang et al., [59] (China)	Detection	295	Automatic	Deep learning	DenseNet	CT images	Accuracy: 92%, sensitivities: 97%, specificity: 0.87
Jaiswal et al., [33] (India)	Detection	2492 (open source)	Automatic	Deep transfer learning	DenseNet201	CT images	Precision: 96.29%, specificity: 96.21%, accuracy: 96.25%
Mahmud et al., [61] (Bangladesh)	Diagnosis	5856	Not mentioned	Deep learning (CNN)	CovXNet	X-ray	Accuracy of multiple classes: 90.2%
Singh et al., [52] (India)	Classification (diagnosis)	Not mentioned	Automatics using CNN	CNN, ANN, and ANFIS	Not mentioned	CT images	Proposed model is compared with CNN, ANFIS, and ANN models and it shows high performance
Ko et al., [45] (Korea)	Diagnosis (differentiate)	3993 patients	Automatic feature extraction	Deep learning (FCONet)	ResNet-50	CT images	Sensitivity: 99.58%, specificity: 100.00%, accuracy: 99.87%
Wu et al., [56] (China)	Screening (diagnosis)	495	Automatic feature extraction	Deep learning (CoroNet)	VGG-19	CT images	Accuracy: 76.0%, sensitivity: 81.1%, specificity: 61.15%
Vaid et al., [38] (Canada)	Detection	181	Automatic feature extraction	Deep learning (CoroNet)	VGG-19	X-ray	Accuracy: 96.3%
Ucar & Korkmaz, [54] (Turkey)	Classification (diagnosis)	Public	Automatic feature extraction	CNN	Deep Bayes SqueezeNet	X-ray	Accuracy for overall class: 98.3%
Toğaçar et al, [53] (Turkey)	Diagnosis	Two open sources (*n* = 295)	Automatic feature extraction	Deep learning (CoroNet)	SqueezeNet and MobileNet	X-ray	Classification rate: 99.27%
Khan et al., [57] (India)	Detection and diagnosis	Two datasets (*n* = 1300)	Automatic feature extraction	Deep learning (CoroNet)	Xception	X-ray	Accuracy: 89.6%
Wu et al., [56] (China)	Screening (diagnosis)	495	Automatic feature extraction	Deep learning (CNN)	ResNet-50	CT images	Accuracy: 0.819%, sensitivity: 0.760%, specificity: 0.811%
Yi et al., [40] (USA)	Classification (detection)	88	Automatic feature extraction	Deep learning (CNN)	Not mentioned	X-ray	Sensitivity: 89%
Martínez et al., [64] (Columbia)	Detection	240	Automatic feature extraction	CNN	NASNet^2^	X-ray	Accuracy: 97%
Das et al., [43] (India)	Screening (diagnosis)	6845	Automatic feature extraction	Deep learning (CNN)	Truncated inception net	X-ray	Sensitivity: 88%, specificity: 100%
Hasan et al., [44] (Iraq)	Diagnosis (classification)	321	Q-deformed entropy feature extraction	Deep transfer learning	LSTM neural network classifier	CT images	Accuracy: 99.68%
Pathak et al., [65] (India)	Classification (detection)	852	Automatic feature extraction	Transfer learning technique	ResNet-50	CT images	Accuracy: 93.01%
Waheed et al., [39] (India)	Detection	1124	Automatic feature extraction	GAN (CovidGAN)	ACGAN^3^, VGG-16	X-ray	Accuracy: 95%, sensitivity: 90%, specificity: 97%
Pereira et al., [49] (Brazil)	Diagnosis (classification)	1144	Automatic feature extraction	Deep learning (CNN)	Inception-V3	X-ray	F1 score: 89%
Mei et al., [48] (USA)	Diagnosis	905	Automatic feature extraction	Deep learning (CoroNet)	Inception_ResNet_V2	CT images	Correctly identifying 17 of 25 (68%) patients, whereas radiologists classified all of these patients as COVID-19 negative
Brunese et al., [31] (Italy)	Detection and diagnosis	6523	Automatic feature extraction	Deep learning (CoroNet)	VGG-16	X-ray	Accuracy: 96.3%
Apostolopoulos et al., [29] (Greece)	Detection	455	Automatic feature extraction	Deep learning (CoroNet)	MobileNetV2	X-ray	Sensitivity: 97.36%, specificity: 99.42%, accuracy: 99.18%
Elaziz et al., [60] (Egypt)	Detection	2 databases (open source)	FrMEMs^4^	Deep learning (CoroNet)	MobileNet	X-ray	Accuracy for first dataset: 96.09%, accuracy for second dataset: 98.09%

^1^Bidirectional generative adversarial network. ^2^Neural architecture search network. ^3^Auxiliary classifier generative adversarial network. ^4^Fractional multichannel exponent moments (FrMEMs).
