# Corrigendum to “Deep Learning in the Detection and Diagnosis of COVID-19 Using Radiology Modalities: A Systematic Review”

**DOI:** 10.1155/2021/9868517

**Published:** 2021-10-25

**Authors:** Mustafa Ghaderzadeh, Farkhondeh Asadi

**Affiliations:** ^1^Student Research Committee, Department and Faculty of Health Information Technology and Management, School of Allied Medical Sciences, Shahid Beheshti University of Medical Sciences, Tehran, Iran; ^2^Department of Health Information Technology and Management, School of Allied Medical Sciences, Shahid Beheshti University of Medical Sciences, Tehran, Iran

In the article titled “Deep Learning in the Detection and Diagnosis of COVID-19 Using Radiology Modalities: A Systematic Review” [1], the Acknowledgements section should be corrected as follows:

“This study is based on project no. 1399/61288 at the Student Research Committee, Shahid Beheshti University of Medical Sciences, Tehran, Iran. The authors appreciate the Student Research Committee and Research & Technology Chancellor at Shahid Beheshti University of Medical Sciences for their financial support of this study.”
